# Construction and validation of a prognostic model based on autophagy-related genes for hepatocellular carcinoma in the Asian population

**DOI:** 10.1186/s12864-023-09367-5

**Published:** 2023-06-27

**Authors:** Yanjie Wang, Sijia Huang, Yingtian Zhang, Yaping Cheng, Liya Dai, Wenwen Gao, Zhengyang Feng, Jialong Tao, Yusong Zhang

**Affiliations:** Department of Oncology, The Second Affiliated Hospital of Soochow University, San Xiang Road No.1055, Suzhou, Jiangsu Province 215004 People’s Republic of China

**Keywords:** Hepatocellular carcinoma, Autophagy-related gene, Prognostic model, The Cancer Genome Atlas database

## Abstract

**Background and objective:**

Hepatocellular carcinoma (HCC), which has a complex pathogenesis and poor prognosis, is one of the most common malignancies worldwide. Hepatitis virus B infection is the most common cause of HCC in Asian patients. Autophagy is the process of digestion and degradation, and studies have shown that autophagy-associated effects are closely related to the development of HCC. In this study, we aimed to construct a prognostic model based on autophagy-related genes (ARGs) for the Asian HCC population to provide new ideas for the clinical management of HCC in the Asian population.

**Methods:**

The clinical information and transcriptome data of Asian patients with HCC were downloaded from The Cancer Genome Atlas (TCGA) database, and 206 ARGs were downloaded from the human autophagy database (HADB). We performed differential and Cox regression analyses to construct a risk score model. The accuracy of the model was validated by using the Kaplan–Meier (K–M) survival curve, receiver operating characteristic (ROC) curve, and univariate and multivariate Cox independent prognostic analyses. The results Thirteen ARGs that were significantly associated with prognosis were finally identified by univariate and multivariate Cox regression analyses. The K–M survival curves showed that the survival rate of the low-risk group was significantly higher than that of the high-risk group (*p* < 0.001), and the multi-indicator ROC curves further demonstrated the predictive ability of the model (AUC = 0.877).

**Conclusion:**

The risk score model based on ARGs was effective in predicting the prognosis of Asian patients with HCC.

## Introduction

Liver cancer is the sixth most common cancer worldwide and has the fourth highest mortality rate among cancers. Hepatocellular carcinoma (HCC) is the most common form of liver cancer and accounts for ~ 90% of liver cancer cases [1]. The incidence and mortality rates of HCC are highest in East Asia and Africa. HCC is expected to become the third leading cause of death due to cancer by 2030 [2]. The etiology of HCC is regionally variable, with hepatitis B virus being the leading cause of HCC in most of Asia (except Japan), South America, and Africa. Hepatitis C virus is the major cause of HCC in Western Europe, North America, and Japan, and alcohol consumption is the main cause of HCC in Central and Eastern Europe [3].

The pathophysiology of HCC is a complex multistage process in which the interaction of multiple factors is the starting point for the malignant transformation of hepatocytes and the development of HCC. Studies have shown that autophagy-related pathways are involved in the development of HCC [4, 5]. Autophagy is the process of transporting damaged, degenerated, or senescent proteins and organelles from cells to lysosomes for digestion and degradation. Autophagy plays a double-edged role in tumors. Under normal physiological conditions, cellular autophagy is beneficial for maintaining a self-stable state, preventing the accumulation of damaged proteins and organelles and inhibiting cellular carcinogenesis. However, once a tumor is formed, cellular autophagy provides rich nutrition to cancer cells and promotes tumor growth [6].

The diagnosis of HCC currently relies on two main methods: imaging and histopathology. Further testing is required for high-risk individuals with suspected liver nodules or abnormal serum AFP levels. On CT scan or MRI, HCC lesions are brighter than the surrounding liver in the arterial phase and less bright than the surrounding parenchyma in the venous and delayed phases [7]. This phenomenon has a sensitivity of 89% and a specificity of 96% for HCC diagnosis [8]. However, there are some tumors with atypical imaging presentations, so a biopsy is recommended. The sensitivity of a biopsy is ~ 70% [9]. In recent years, studies have also shown the significance of liquid biopsy in the diagnosis of HCC, and some scientists have worked to find new biomarkers for the early detection and diagnosis of HCC to improve the prognosis of HCC patients [10].

For patients with early-stage HCC, surgical intervention, including resection, transplantation and local ablation, is recommended in principle. The preferred treatment option for patients with intermediate-stage HCC is TACE, and patients with advanced HCC are recommended to first receive systemic therapy [11]. Nevertheless, the prognosis of patients with HCC remains unideal. The prognosis of HCC is mainly assessed on the basis of staging, such as BCLC and TNM staging. However, the results of staging are unsatisfactory, and tools that can accurately assess HCC remain lacking. Therefore, making prognostic predictions with increased accuracy is crucial for the clinical management of patients with HCC.

In consideration of the appreciable regional differences in the etiology of HCC and the fact that East Asia is the region with the highest incidence of HCC, we selected the Asian population as our study target and constructed a risk score prognostic model based on autophagy-related genes (ARGs) for the Asian HCC population through the analysis of the transcriptome and clinical data of the Asian HCC population in The Cancer Genome Atlas (TCGA) database. Our work may provide novel ideas for the discovery of prognostic assessment methods, new therapeutic targets, and biomarkers of HCC in the Asian population.

**Fig. 1:**
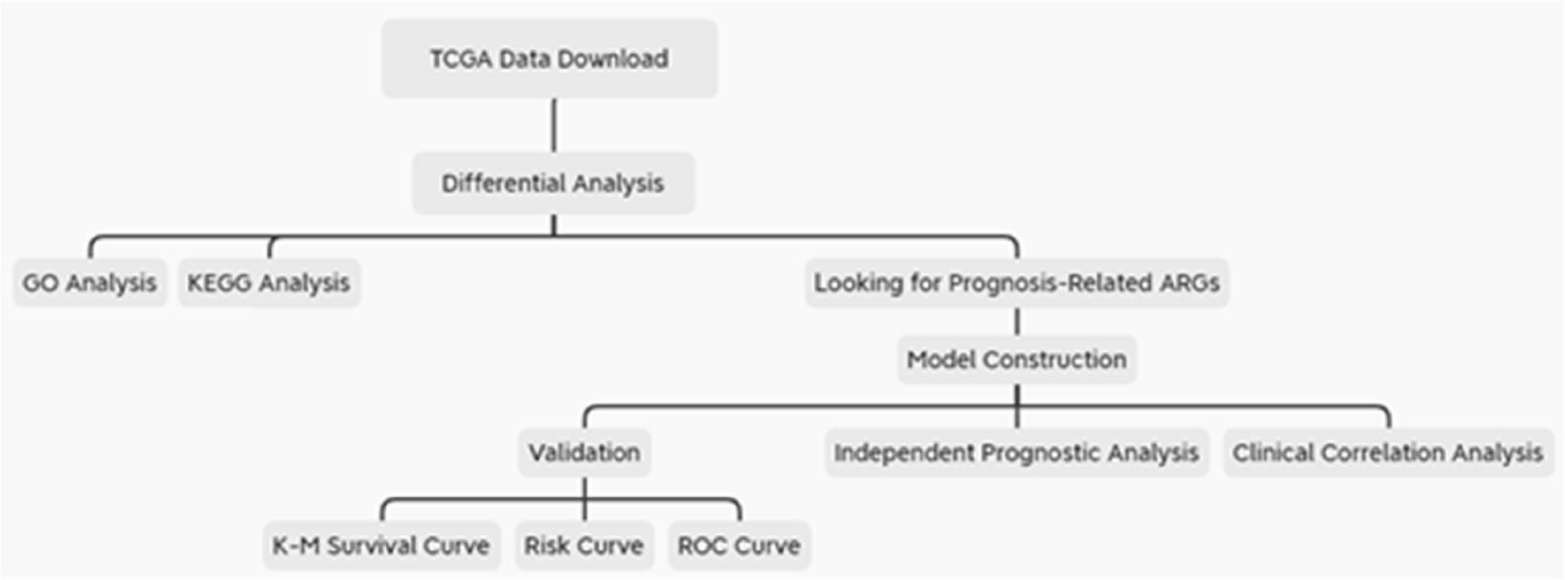
Diagram of the study workflow

The workflow of this study is shown in Fig. 1.

## Data acquisition and analysis methods

### Data acquisition

In May 2022, the transcriptome sequencing data and clinical information of 161 Asian patients with HCC were downloaded from the TCGA–LIHC project from the TCGA website (https://portal.gdc.cancer.gov/). The data included the transcriptome data, which contained the sequencing information of six normal samples and 160 tumor samples, and the clinical information detailed in Table 1. Human ARGs were downloaded from the HADB website (http://www.autophagy.lu/clustering/index.html, last update time: 2021–12-3), and a total of 206 ARGs were obtained.

**Table 1:** Clinical features of 106 Asian patients with HCC

Clinical Feature	Classification	Number	Proportion
Age	≤ 65	125	77.64%
	> 65	35	21.74%
	Unknown	1	0.62%
Gender	Female	34	21.12%
	Male	127	78.88%
Grade	G1	14	8.70%
	G2	64	39.75%
	G3	71	44.10%
	G4	12	7.45%
Stage	I	81	50.31%
	II	36	22.36%
	III	41	25.47%
	IV	1	0.62%
	Unknown	2	1.24%
TNM			
T	T1	82	50.93%
	T2	36	22.36%
	T3	37	22.98%
	T4	6	3.73%
N	N0	149	92.55%
	N1	1	0.62%
	NX	11	6.83%
M	M0	153	95.03%
	M1	1	0.62%
	MX	7	4.35%
Survival State	Survival	117	72.67%
	Dead	44	27.33%

### Differential expression and enrichment analyses

#### Finding differential ARGs

After obtaining ARG information, ARG expression was extracted from the transcriptome data, and the genes with zero expression in normal and tumor samples were removed. Next, differential analysis was performed by applying the Wilcox test in R language. The filtering conditions for filtering out differential ARGs were set as follows: fdr ≤ 0.05 (where the fdr value represents the false discovery rate), | logFC|≥ 1 (logFC=  ${log}_2\frac{Mean\;value\;of\;tumor\;sample\;gene\;expression}{Mean\;value\;of\;normal\;sample\;gene\;expression}$).

#### Enrichment analysis

Gene Ontology (GO) and Kyoto Encyclopedia of Genes and Genomes (KEGG) enrichment analyses were performed on the obtained differential ARGs by using the enrichplot package in R language on the basis of the GO and KEGG databases. The GOs and KEGGs wherein each differential gene was located were identified in the backend database org.Hs.eg.db. Next, the differentially expressed genes were analyzed for enrichment by using Fisher's exact test. Then, their *p* values were calculated for testing. GOs and KEGGs that met the conditions were screened in accordance with *p* ≤ 0.05 and q ≤ 0.05 (q-values are corrected *p* values). The results of GO enrichment analysis were classified in accordance with biological process (BP), cellular component (CC), and molecular function (MF).

### Prognostic model construction and validation

#### Finding prognosis-related autophagy genes

The survival package in R language was used to find prognosis-related ARGs by combining differential ARG expression with the survival time and survival status from the clinical data obtained from TCGA. Cox analysis was performed to compare survival time and survival status with ARG expression. The screening condition was set to *p* ≤ 0.05.

#### Construction and validation of the ARG-based prognostic model

The survival package in R was used to construct the ARG-based prognostic model. First, the survival time and survival state were read by the coxph function, and the Cox model was initially constructed. Then, the model was optimized by removing the genes with high correlation by using the step function. Next, the optimized model parameters were obtained by using the summary function, and the coefficients (coef), HR values, 95% CI of HR values, and *p* values were all output. After obtaining the model coefficients, the predict function was used to calculate the risk score of the patients (risk score = $\sum_{i}^{n}{coef}_{i}\times {Exp}_{i}$, where Exp represents gene expression) and the median risk score based on the constructed prognostic model. The patients were divided into high- and low-risk groups in accordance with median values.

The difference analysis of the high- and low-risk groups was performed by using the survdiff function in the survival package. Kaplan–Meier (K–M) survival curves were plotted by using the ggsurvplot function to compare the survival conditions of the high- and low-risk groups. The survivalROC package was applied to plot the receiver operating characteristic (ROC) curve, and the accuracy of the prognostic model was assessed by using the area under the ROC curve (AUC). The plot function in the pheatmap package was used to plot the risk curve, and univariate and multivariate Cox independent prognostic analyses were performed to evaluate whether the risk score can be regarded as an independent prognostic factor.

### Clinical correlation analysis

All clinical features from the clinical data obtained from the TCGA database were divided into two groups as follows: age: ≤ 65 and > 65 years, sex: male and female, pathological grade: G1–2 and G3–4, pathological stage: stages I–II and stages III–IV, T stage: T1–2 and T3–4, M stage: M0 and 1 (cases with distant metastases), and N stage: N0 and 1 (cases with lymph node metastasis). The ARGs involved in the model, as well as the risk score, were compared with each clinical trait and analyzed for correlation by using the beeswarm package in R. A *p* value less than 0.05 indicated that the ARG or risk score was associated with a specific clinical trait.

### Statistical methods

All analyses were completed by using R version 4.1.0. Difference analysis was conducted with the Wilcox test, and the enrichment analysis of differential ARGs was performed by using Fisher's exact test. Cox regression analysis was employed for prognostic model construction, and K–M survival curves, as well as the log-rank test, were used to assess the survival differences between patients in the high- and low-risk groups. In addition, univariate and multivariate Cox independent prognostic analyses and ROC curves were applied to assess the predictive ability of the model.

## Results

### Differential ARGs

**Fig. 2:**
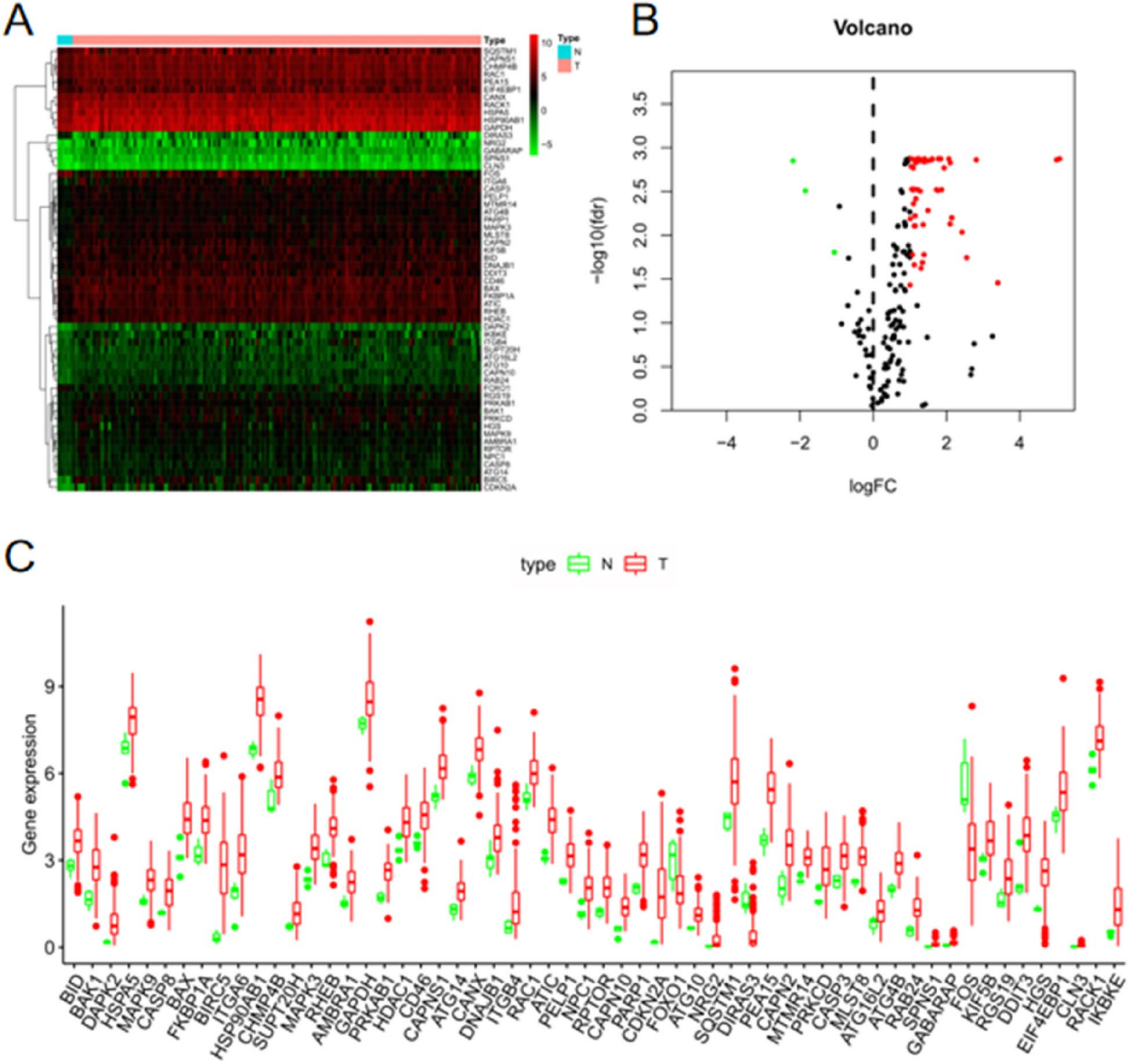
Expression of differential ARGs in normal and tumor samples. **A** Heatmap of the expression of 58 differential ARGs. Horizontal coordinates are sample names (the blue group is normal samples, and the red group is tumor samples). Vertical coordinates are the gene names of differential ARGs. Green represents low expression, black indicates intermediate expression, and red designates high expression. **B** Volcano plot of 58 differential ARGs. The horizontal coordinate is LogFC, and the vertical coordinate is − (fdr). A high absolute LogFC value indicates that a gene is differentially expressed in normal and tumor samples. Green dots represent genes that are downregulated in tumor samples. and red reflects upregulated genes. Black dots indicate genes that are not differentially expressed. **C** Box plot of 58 differential ARGs. The horizontal coordinate is differential ARGs, and the vertical coordinate is gene expression. Green represents the normal group, and red represents the tumor group

A total of 58 differential ARGs were screened through differential analysis using the WilcoxTest (Fig. 2A–B). The box plot in Fig. 2C shows the expression of differential ARGs between tumor and normal samples.

### Enrichment analysis

**Fig. 3:**
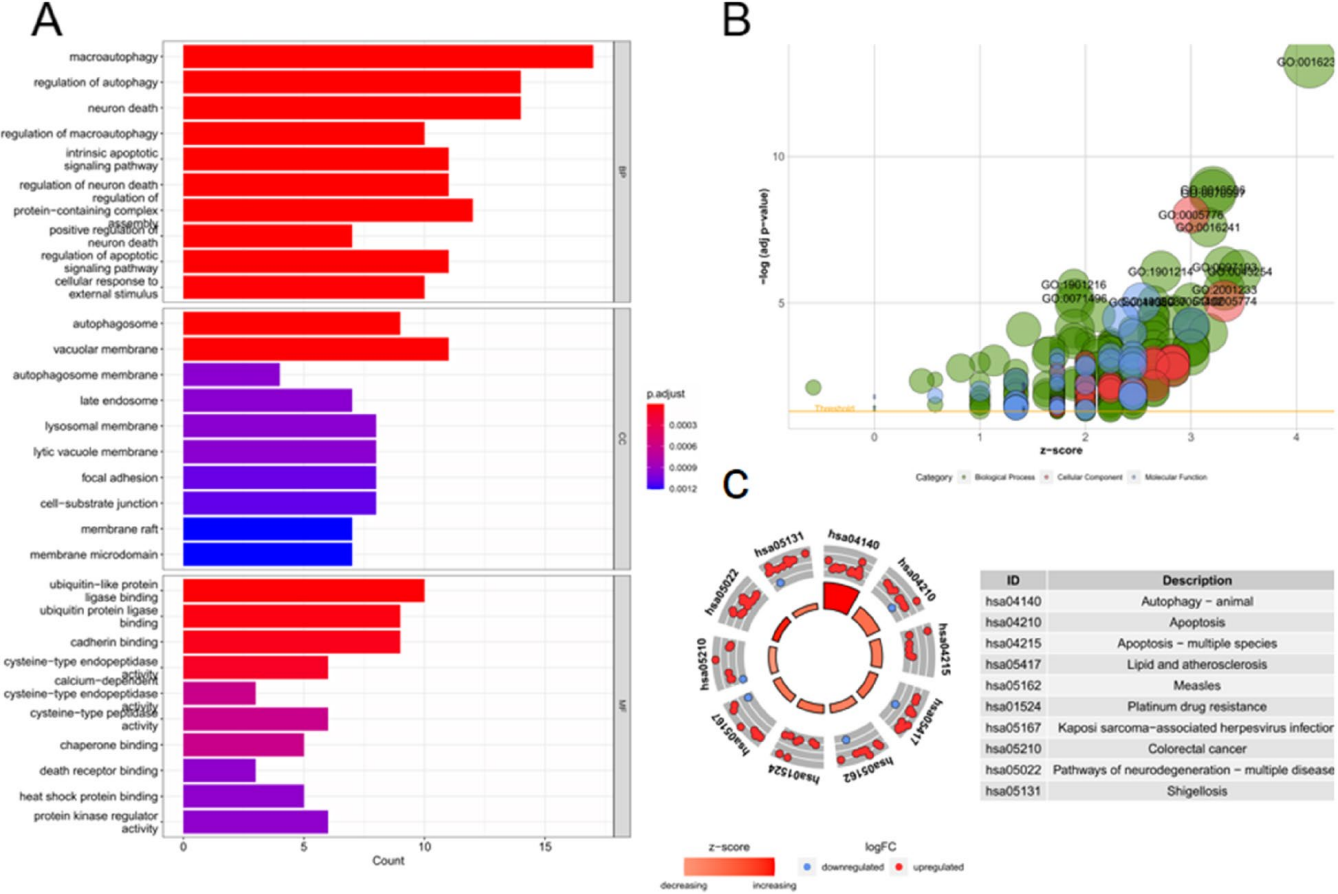
GO and KEGG enrichment results of differential ARGs. **A** Bar chart of the GO enrichment analysis of differential ARGs. The horizontal coordinate is the number of genes enriched in each GO, and the vertical coordinate is the GO name, which is divided into three categories: BP, CC, and MF. The length of the bar represents the number of genes, and the color represents the degree of enrichment. The differential ARGs were enriched in macroautophagy, the regulation of autophagy, and other functions. **B** Bubble plot of the GO analysis of differential ARGs. The horizontal coordinate is the Z score. (Z score = $\sqrt{\frac{Number\;of\;upregulated\;genes\;in\;a\;GO\;minus\;the\;number\;of\;downregulated\;genes}{\mathrm{Total\;number\;of\;genes\;enriched\;on\;this\;GO}}}$). A Z score > 0 indicates that more upregulated genes are enriched in a specific GO, whereas a Z score < 0 indicates the opposite. The vertical coordinate represents − log(adjp-value). **C** Circles of differential ARGs obtained through KEGG enrichment analysis. The inner circle represents the Z score value. The redder the color, the more upregulated genes are enriched in that pathway, whereas the bluer the color, the more downregulated genes are enriched in that pathway. The middle circle shows the number of up- and downregulated genes in each pathway, and the outer circle shows the KEGG IDs. Differential ARGs are highly enriched in autophagy–animal and apoptosis

GO and KEGG enrichment analyses were performed on the 58 screened differential ARGs. Figure 3A–B show the top 30 results of GO enrichment analysis classified into BP, CC, and MF. The GO function enrichment results showed that the differentially expressed ARGs were mainly enriched in functions such as macroautophagy and the regulation of autophagy. KEGG enrichment analysis demonstrated that the differential ARGs were mainly enriched in pathways such as autophagy–animal and apoptosis (Fig. 3C) [12–14].

### Prognostic model construction and validation

#### Prognostic model construction

**Fig. 4:**
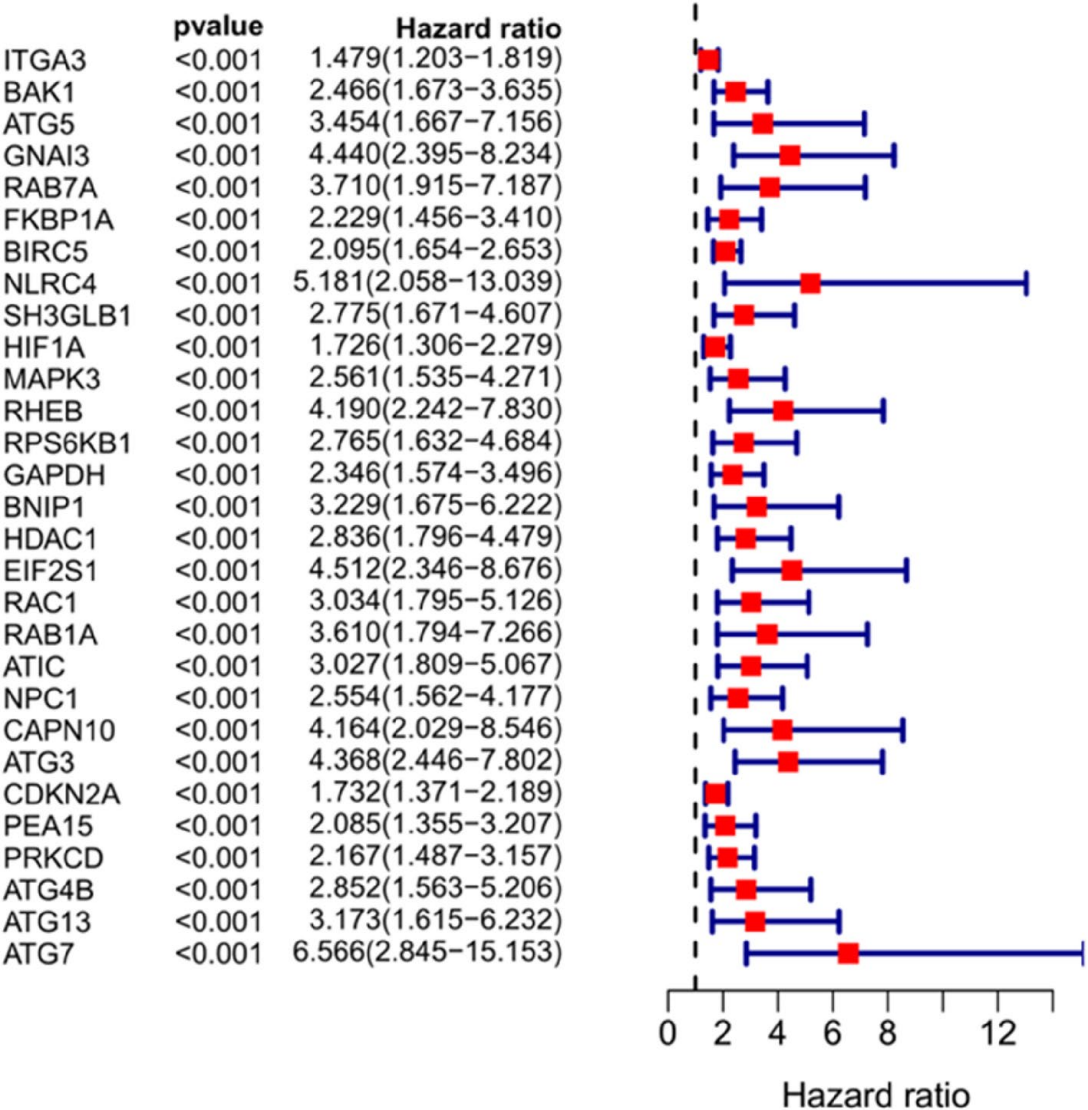
Forest plot of the 29 prognosis-related ARGs

**Fig. 5:**
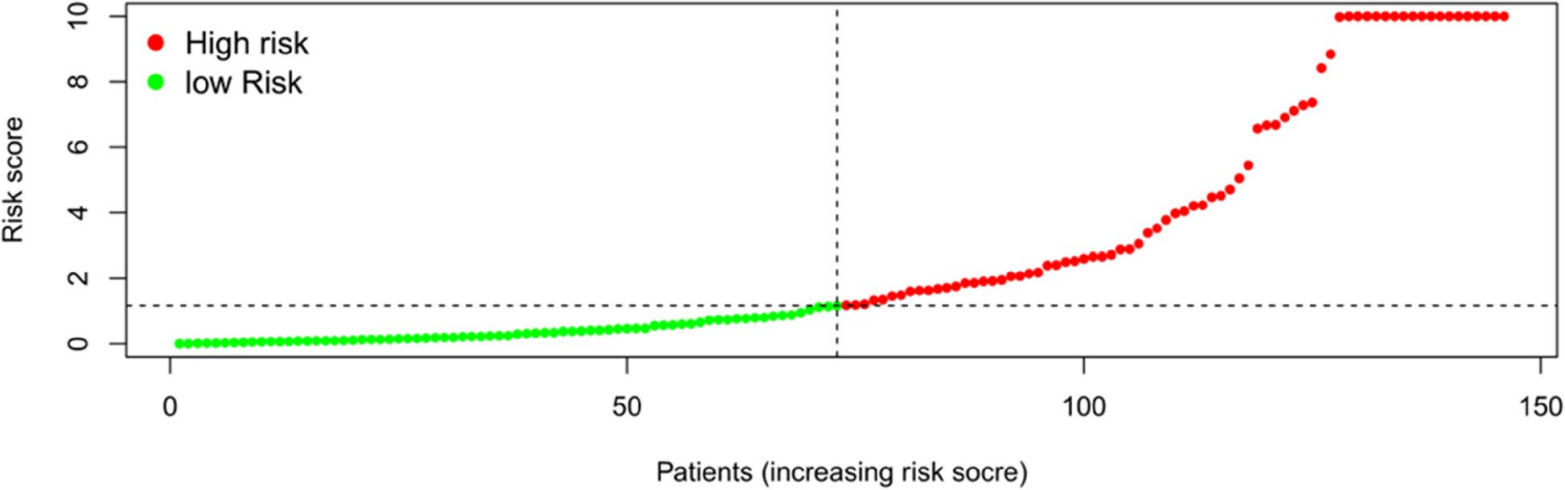
Risk score curve of 161 Asian patients with HCC. The risk scores increase from left to right, with the median value at the dotted line. The green line on the left represents low-risk patients, whereas the red line on the right indicates high-risk patients

A total of 29 prognosis-related ARGs with *p* < 0.001 were screened via single-factor Cox analysis (Fig. 4). An additional 13 significant prognosis-related ARGs were screened by multifactor Cox regression analysis. They included G protein subunit alpha I3 (GNAI3); FKBP prolyl isomerase 1A (FKBP1A); baculoviral IAP repeat-containing 5 (BIRC5); SH_3_ domain-containing GRB2 like; endophilin B1 (SH3GLB1); hypoxia inducible factor 1 subunit alpha (HIF1A); RAS homolog; MTORC1 binding (RHEB); eukaryotic translation initiation factor 2 subunit alpha (EIF2S1); member RAS oncogene family RAB1A (RAB1A); 5-aminoimidazole-4-carboxamide ribonucleotide formyltransferase/IMP cyclohydrolase (ATIC); NPC intracellular cholesterol transporter 1 (NPC1); protein kinase C delta (PRKCD); autophagy-related 4B cysteine peptidase (ATG4B); and CLN3 lysosomal/endosomal transmembrane protein, Battenin (CLN3) (Table 2). The risk score model for predicting prognosis was constructed by applying the above ARGs: risk score = (1.663674611) × Exp_GNAI3_ + (− 0.6607235) × Exp_FKBP1A_ + (0.801978919) × Exp_BIRC5_ + (− 1.24003891) × Exp_SH3GLB1_ + (− 0.558780456) × Exp_HIF1A_ + (0.743003202) × Exp_RHEB_ + (0.994004247) × Exp_EIF2S1_ + (1.300632) × Exp_RAB1A_ + (0.869938532) × Exp_ATIC_ + (− 0.730288207) × Exp_NPC1_ + (0.708888933) × Exp_PRKCD_ + (− 1.092151453) × Exp_ATG4B_ + (10.04890334) × Exp_CLN3_. The risk scores of the patients were calculated by using the model, and the patients were divided into high- and low-risk groups in accordance with median values (Fig. 5).

**Table 2:** Thirteen ARGs involved in the construction of the risk score prognostic model

id	coef	HR	HR.95 L	HR.95H	*P* value
GNAI3	1.663674611	5.278672319	1.799225752	15.48687341	0.002449315
FKBP1A	-0.6607235	0.516477528	0.23218198	1.148879155	0.105289371
BIRC5	0.801978919	2.229949454	1.405555164	3.537872219	0.000660152
SH3GLB1	-1.24003891	0.289372958	0.1049318	0.79801079	0.016578861
HIF1A	-0.558780456	0.571906104	0.322267562	1.014922475	0.056216872
RHEB	0.743003202	2.102239494	0.827849879	5.338420655	0.118138896
EIF2S1	0.994004247	2.702032444	0.966307011	7.555548338	0.058140623
RAB1A	1.300632	3.671616397	1.141455922	11.81015114	0.02911576
ATIC	0.869938532	2.386764141	1.179449082	4.829918602	0.015568299
NPC1	-0.730288207	0.481770121	0.203889049	1.138376243	0.096000979
PRKCD	0.708888933	2.031732613	1.131692333	3.647579198	0.017580932
ATG4B	-1.092151453	0.335493917	0.127267022	0.88440954	0.027220774
CLN3	10.04890334	23,130.40663	1.402742856	381,406,833.4	0.042532427

#### Reliability analysis of the risk score prognostic model

**Fig. 6:**
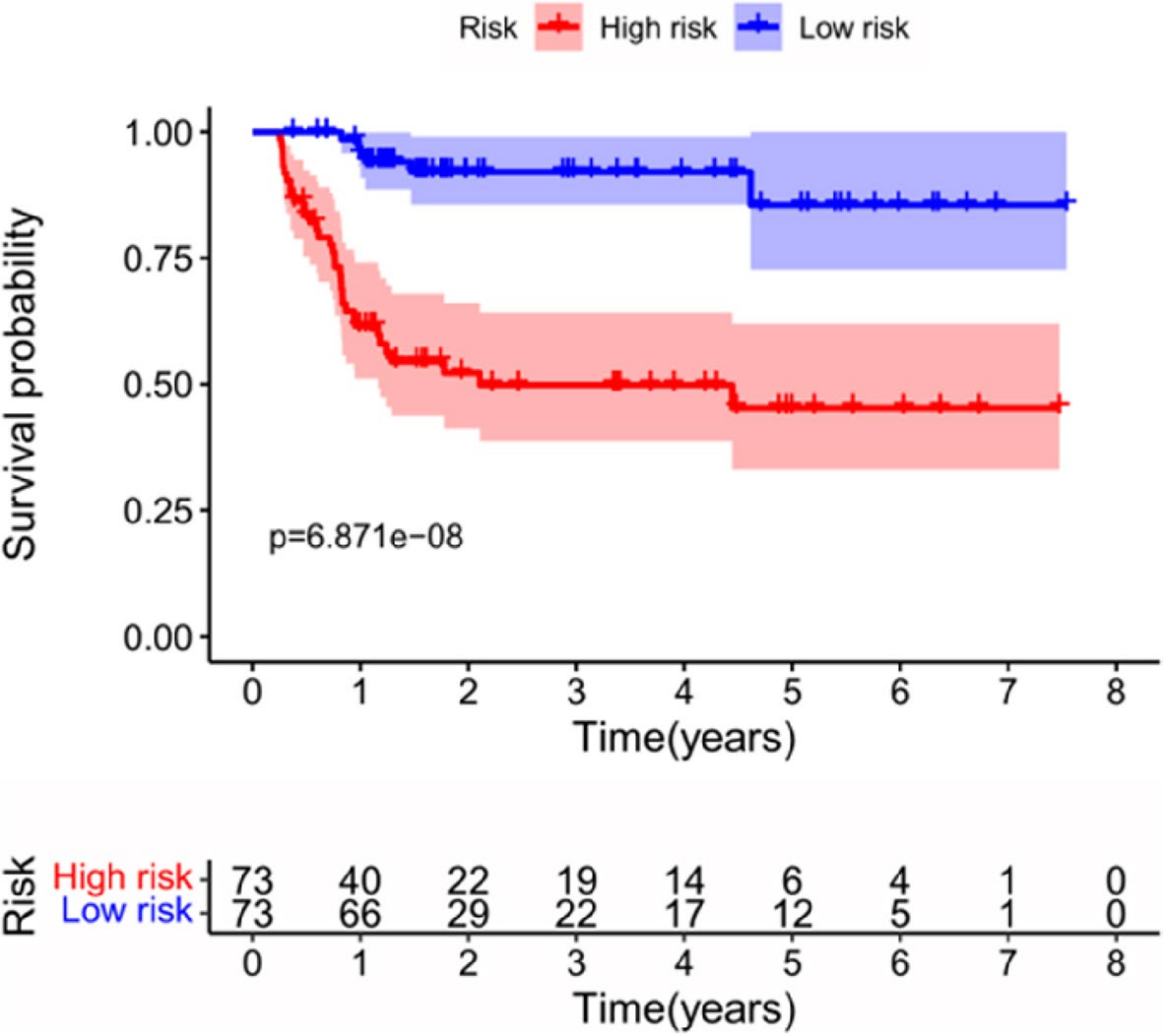
K–M survival curve of 161 Asian patients with HCC and primary liver cancer at different risk levels. The red curve represents the high-risk group, the blue curve represents the low-risk group, and the shading represents the 95% confidence interval. The survival rate and survival time were significantly higher in the low-risk group than in the high-risk group (*p* < 0.05)

**Fig. 7:**
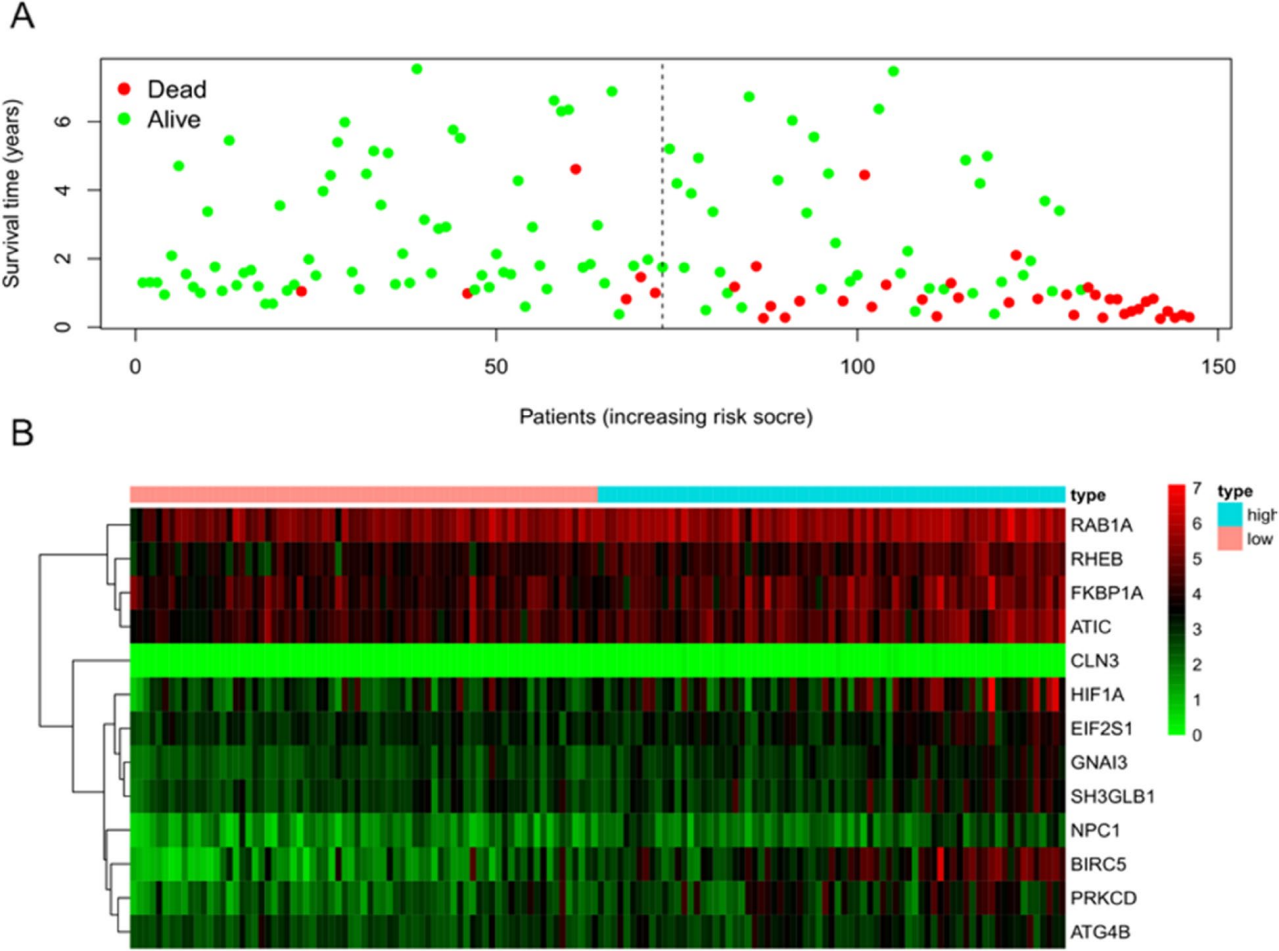
Relationship of the risk score with survival time and gene expression. **A** Survival time. Red dots represent death, and green dots represent survival. With the increase in risk score, the survival time decreases, and the mortality rate gradually increases. **B** Heatmap of the relationship between the risk score and the expression of the 13 ARGs involved in the construction of the prognostic model. The expression of the 13 ARGs was higher in the high-risk group than in the low-risk group

**Fig. 8:**
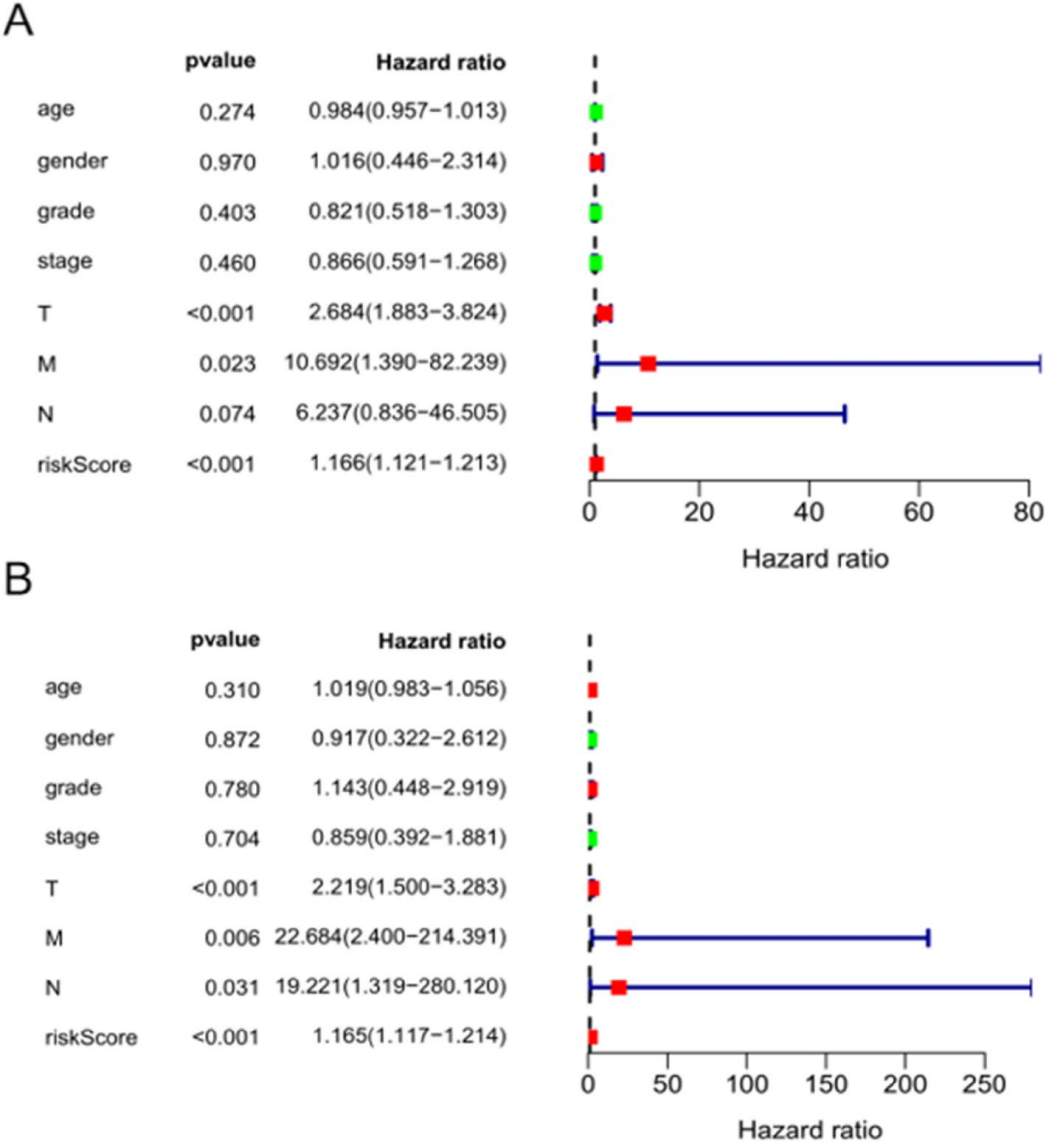
Forest plots of Cox independent prognostic analysis. **A** Univariate Cox independent prognostic analysis **B** Multivariate Cox independent prognostic analysis The *p* values of the T stage, M stage, and risk score are less than 0.05 in univariate and multivariate Cox independent prognostic analyses, indicating that all of these indices can be regarded as independent prognostic factors of primary liver cancer

**Fig. 9:**
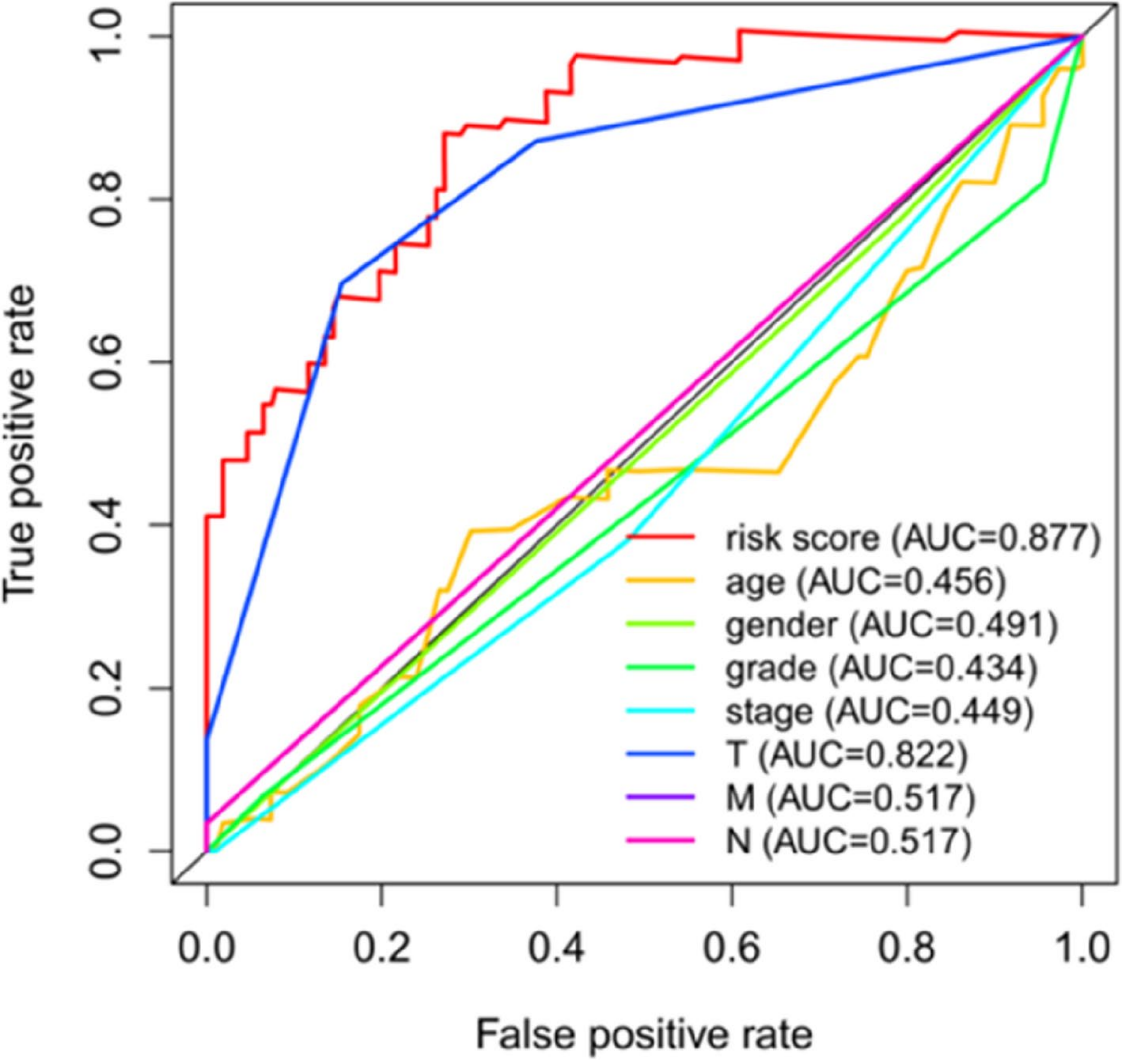
Multi-indicator ROC curve. The AUC value of the risk score was significantly higher than that of other clinical features

K–M survival curves were plotted to compare the survival time of the high- and low-risk groups. The survival rate of the low-risk group (60.6%, 95% CI: 48.5%–75.6%) was higher than that of the high-risk group (35.9%, 95% CI: 25.01%–51.40%) (Fig. 6). The mortality rate increased progressively with the increase in risk scores (Fig. 7A), indicating that the high-risk group had a poor prognosis. Figure 7B shows the heatmap of the 13 model ARGs in the high- and low-risk groups. The expression of these 13 ARGs was higher in the high-risk group than in the low-risk group. Univariate and multivariate Cox independent prognostic analyses found that the *p* values of the risk score prognostic model were less than 0.05, demonstrating that the risk score calculated by this model can be regarded as an independent prognostic factor for evaluating the prognosis of patients with primary liver cancer (Fig. 8A–B). Multi-indicator ROC curves were used to compare the predictive ability of the risk score (AUC = 0.877) with that of other clinical traits, such as age (AUC = 0.456), sex (AUC = 0.491), pathological grade (AUC = 0.434), pathological stage (AUC = 0.449), and TNM stage (AUC_T_ = 0.822, AUC_M_ = 0.517, AUC_N_ = 0.517). The significantly higher AUC value of the risk score than that of the other clinical traits indicated that the ability of the risk score to predict prognosis was better than that of the remaining clinical traits (Fig. 9).

### Clinical correlation analysis

**Fig. 10:**
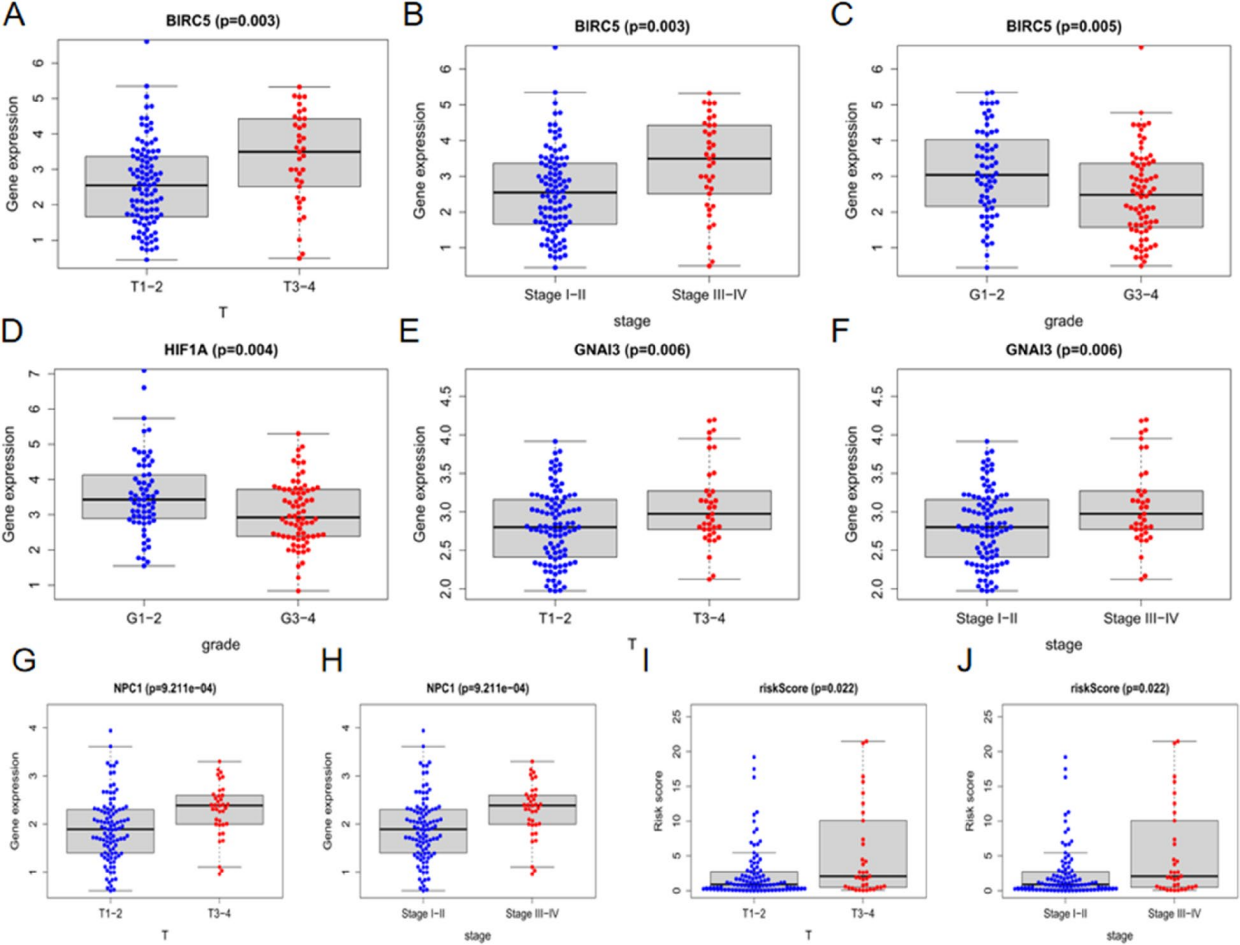
Clinical correlation analysis. The expression levels of BIRC5, GNAI3, and NPC1 and risk scores were lower in the T1–2 group than in the T3-4 group. The expression levels of BIRC5, GNAI3, and NPC1 and risk scores were lower in the stage I–II group than in the stage III–IV group. The expression levels of BIRC5 and HIF1A were higher in the G1–2 group than in the G3–4 group. The *p* values are less than 0.01, indicating statistical significance

The 13 ARGs involved in the construction of the model, as well as the risk scores, were compared with each clinical trait. The results showed that BIRC5 was closely correlated with T-stage and pathological grade and stage (Fig. 10A–C); HIF1A was highly related to pathological grade (Fig. 10D); and GNAI3 and NPC1 together with the risk score were strongly related to the T and pathological stages (Fig. 10E–J).

## Discussion

Autophagy is an ancient evolutionary mechanism that prompts lysosomes to degrade excess or potentially dangerous cellular content, such as damaged, degenerated or senescent proteins, and organelles within cells [6]. Although autophagy mainly plays an adaptive role in protecting organisms from various pathological changes, it can also be detrimental in specific environments.

A growing body of evidence shows that autophagy is closely associated with the development of HCC. The physical interaction of the autophagy adaptor p62 with the Nrf2 inhibitor Keap1 leads to reprogramming of the metabolic and stress response pathways of proliferating HCC cells [4]. HIF-1α-induced YTHDF1 expression is associated with HCC progression by promoting the translation of the autophagy-related genes ATG2A and ATG14 in a m6A-dependent manner [5]. However, the relationship between autophagy and the prognosis of HCC remains unclear.

In this study, by analyzing the clinical and transcriptome data of HCC in the Asian population downloaded from the TCGA database and human ARGs, 13 significant prognosis-related ARGs, namely, GNAI3, FKBP1A, BIRC5, SH3GLB1, HIF1A, RHEB, EIF2S1 RAB1A, ATIC, NPC1, PRKCD, ATG4B, and CLN3, were screened via multifactorial Cox regression analysis. We constructed a risk score prognostic model based on the above 13 genes. Subsequently, by plotting K–M survival curves, we found that the survival rate of the group with low risk scores was significantly higher than that of the group with high risk scores (*p* < 0.001). Univariate and multivariate Cox independent prognostic analyses revealed that the risk score could be used as an independent prognostic factor for HCC. The ROC curves showed that the AUC value of the risk score (AUC = 0.877) was significantly higher than that of other clinical traits, indicating that the ability of the risk score model to predict prognosis was better than that of other models.

A few of the 13 ARGs in our risk score model have been reported to be related to HCC. GNAI3 is downregulated in HCC relative to the noncancerous liver. Transwell assays indicated that GNAI3 inhibits HCC cell migration and invasion [15]. Starvation induction can promote the expression of EIF2S1 and P-EIF2S1 in HCC, and the increased expression of EIF2S1 may affect the invasion and metastasis ability of HCC [16]. ATIC inhibits the activation of adenosine monophosphate-activated protein kinase, thereby activating MTOR-S6K1-S6 signaling and supporting the growth and motility of HCC cells [17].

Although most of the remaining ARGs have not been reported to be associated with HCC thus far, some studies suggest that they may have some connections to other cancers. FKBP1A can be regulated by SNHG15, which is closely related to the occurrence of prostate cancer [18]. HIF1A can act as a tumor suppressor by preventing the expression of PPP1R1B and the subsequent degradation of the p53 protein in pancreatic cancer cells [19]. ATG4B expression is highly elevated in human epidermal growth factor receptor 2-positive breast cancer and colorectal cancer [20, 21]. BIRC5 has been characterized in several solid and hematologic tumors [22]. The allelic loss of SH3GLB1 may play a crucial role in the premalignant stage [23]. RAB1A overexpression promotes cancer cell migration, invasion, and metastasis by activating JAK1/STAT6 signaling [24].

The four ARGs RHEB, NPC1, PRK3D, and CLN3 have not been reported in previous studies on HCC or other cancers. RHEB is related to GTP binding and GTPase activity https://www.genecards.org/cgi-bin/carddisp.pl?gene=RHEB&keywords=rheb, and CLN3 is associated with unfolded protein binding https://www.genecards.org/cgi-bin/carddisp.pl?gene=CLN3&keywords=CLN3. The loss of NPC1 may play a specific role in neuronal death [25]. PRKCD can inhibit macroautophagy/autophagy by phosphorylating AKT [26].

However, the mechanism by which these 13 ARGs affect HCC prognosis through autophagy-related functions has not been validated in previous studies. Our study suggests that these genes are very likely to influence the prognosis of HCC through autophagy. Further understanding of the function of these ARGs and their role in autophagy may facilitate the development of HCC therapy.

This study has some limitations. First, the study data were obtained from public databases, the sample size was insufficient, and missing data were rare. Second, this study is a retrospective study with a series of biases, and further large-scale prospective and multicenter clinical trials are needed to verify the predictive ability of the model accurately. Third, although the survival analysis and ROC curve validation are excellent, further validation of external datasets, such as the GEO dataset, is still needed. In addition, the role of the 13 ARGs in the pathogenesis and prognosis of HCC remains unclear and must be explored and validated through additional in vitro and in vivo experiments.

## Conclusion

We constructed and validated a prognostic risk score model for HCC in an Asian population. This model, which was based on 13 ARGs, including GNAI3, FKBP1A, BIRC5, SH3GLB1, HIF1A, RHEB, EIF2S1 RAB1A, ATIC, NPC1, PRKCD, ATG4B, and CLN3, can predict the prognosis of HCC. The 13 genes involved in this model have the potential to become new targets for HCC treatment. In the current era of precision medicine, this discovery undoubtedly provides new perspectives for the treatment and clinical management of patients with HCC.

## Data Availability

The datasets analyzed during the current study are available in the TCGA repository [https://portal.gdc.cancer.gov/]. The detailed link and steps for data download are shown below. • Transcriptome data download. Go to the following link, then add all files to cart, and finally download three files from cart: manifest, cart, and metadata. [https://portal.gdc.cancer.gov/repository?facetTab=files&filters=%7B%22op%22%3A%22and%22%2C%22content%22%3A%5B%7B%22op%22%3A%22in%22%2C%22content%22%3A%7B%22field%22%3A%22cases.demographic.race%22%2C%22value%22%3A%5B%22asian%22%5D%7D%7D%2C%7B%22op%22%3A%22in%22%2C%22content%22%3A%7B%22field%22%3A%22cases.primary_site%22%2C%22value%22%3A%5B%22liver%20and%20intrahepatic%20bile%20ducts%22%5D%7D%7D%2C%7B%22op%22%3A%22in%22%2C%22content%22%3A%7B%22field%22%3A%22cases.project.program.name%22%2C%22value%22%3A%5B%22TCGA%22%5D%7D%7D%2C%7B%22op%22%3A%22in%22%2C%22content%22%3A%7B%22field%22%3A%22cases.project.project_id%22%2C%22value%22%3A%5B%22TCGA-LIHC%22%5D%7D%7D%2C%7B%22op%22%3A%22in%22%2C%22content%22%3A%7B%22field%22%3A%22files.data_category%22%2C%22value%22%3A%5B%22transcriptome%20profiling%22%5D%7D%7D%2C%7B%22op%22%3A%22in%22%2C%22content%22%3A%7B%22field%22%3A%22files.data_type%22%2C%22value%22%3A%5B%22Gene%20Expression%20Quantification%22%5D%7D%7D%5D%7D]. If the above link is not directly accessible, go to the data download home page from the following link and follow the steps to filter the information and then download the data: [https://portal.gdc.cancer.gov/repository]. The screening criteria in cases are as follows. [Primary Site: Liver and intrahepatic bile ducts; Project: TCGA-LIHC; Disease type: adenomas and adenocarcinomas; Race: Asian]. The screening criteria in files are as follows. [Data category: transcriptome profiling; Data type: gene expression quantification; Workflow type: STAR-Counts]. After filtering, add all files to cart and download three files from cart: manifest, cart, and metadata. The transcriptome data required for this study can be obtained by the above steps. • Clinical data download Go to the following link, then add all files to cart, and finally download a cart file from cart. [https://portal.gdc.cancer.gov/repository?facetTab=files&filters=%7B%22op%22%3A%22and%22%2C%22content%22%3A%5B%7B%22op%22%3A%22in%22%2C%22content%22%3A%7B%22field%22%3A%22cases.demographic.race%22%2C%22value%22%3A%5B%22asian%22%5D%7D%7D%2C%7B%22op%22%3A%22in%22%2C%22content%22%3A%7B%22field%22%3A%22cases.primary_site%22%2C%22value%22%3A%5B%22liver%20and%20intrahepatic%20bile%20ducts%22%5D%7D%7D%2C%7B%22op%22%3A%22in%22%2C%22content%22%3A%7B%22field%22%3A%22cases.project.program.name%22%2C%22value%22%3A%5B%22TCGA%22%5D%7D%7D%2C%7B%22op%22%3A%22in%22%2C%22content%22%3A%7B%22field%22%3A%22cases.project.project_id%22%2C%22value%22%3A%5B%22TCGA-LIHC%22%5D%7D%7D%2C%7B%22op%22%3A%22in%22%2C%22content%22%3A%7B%22field%22%3A%22files.data_category%22%2C%22value%22%3A%5B%22clinical%22%5D%7D%7D%2C%7B%22op%22%3A%22in%22%2C%22content%22%3A%7B%22field%22%3A%22files.data_format%22%2C%22value%22%3A%5B%22bcr%20xml%22%5D%7D%7D%5D%7D]. If the above link is not directly accessible, go to the data download home page from the following link and follow the steps to filter the information and then download the data: [https://portal.gdc.cancer.gov/repository]. The screening criteria in cases are as follows. [Primary site: liver and intrahepatic bile ducts; Project: TCGA-LIHC; Disease type: adenomas and adenocarcinomas; Race: Asian]. The screening criteria in files are as follows: [Data category: clinical; Data format: bcr xml]. After filtering, all the data were selected and added to the cart, and then a cart file was downloaded from the cart. The clinical data required for this study can be obtained by the above steps. • Additional statement Note that when adding data to the cart, please make sure that the cart is empty with no additional data. In addition, the data for this study were downloaded from the TCGA official website in May 2022, and we cannot exclude that there are changes or updates in the database from May 2022 to the present.

## Abbreviations

HCC: Hepatocellular carcinoma
ARG: Autophagy-related gene
TCGA: The Cancer Genome Atlas database
HADB: Human autophagy database
K-M: Kaplan–Meier
ROC: Receiver operating characteristic
GO: Gene Ontology
KEGG: Kyoto Encyclopedia of Genes and Genomes
BP: Biological process
CC: Cellular component
MF: Molecular function
coef: Coefficients
Exp: Gene expression
AUC: Area under the ROC curve
